# Punitive Social Policy and Vital Inequality

**DOI:** 10.1177/00207314211024895

**Published:** 2021-06-09

**Authors:** Elias Nosrati, Lawrence P. King

**Affiliations:** 1 Merton College, 6396 University of Oxford, Oxford, UK; 2 14707 UMass Amherst, Amherst, MA, USA

**Keywords:** punitive social policy, incarceration, premature mortality, life expectancy, inequality

## Abstract

Geographical inequalities in life and death are among the world's most pronounced in the United States. However, the driving forces behind this macroscopic variation in population health outcomes remain surprisingly understudied, both empirically and theoretically. The present article steps into this breach by assessing a number of theoretically informed hypotheses surrounding the underlying causes of such spatial heterogeneity. Above and beyond a range of usual suspects, such as poverty, unemployment, and ethno-racial disparities, we find that a hitherto neglected *explanans* is prison incarceration. In particular, through the use of previously unavailable county-level panel data and a compound instrumentation technique suited to isolating exogenous treatment variation, high imprisonment rates are shown to substantially increase the population-wide risk of premature death. Our findings contribute to the political economy of population health by relating the rise of the carceral state to the amplification of geographically anchored unequal life chances.

The unequal distribution of disability, disease, and death in the United States is among the most pronounced in the world and, as documented by a number of recent studies,^1^ the health gap between the top and the bottom of the social order is rapidly increasing. A distinctive feature of this inequality is its geographical patterning, as evidenced by large and persistent spatial variation in key outcomes such as life expectancy at birth.^2^ There appears to exist a strong correspondence between social and physical space, yet rigorous empirical studies of such geographical heterogeneity remain scarce. The present article probes the macroscopic forces that might explain this phenomenon and identifies an institution of major social scientific interest—the prison—as an important yet largely neglected determinant of socially constructed unequal life chances of human organisms, or *vital inequality*.^3^ Using previously unavailable panel data at the county level and a novel instrumentation technique, we provide causal evidence that penal expansion has deepened geographically anchored disparities in survival and well-being. Our investigation unfolds in 3 principal steps. First, we survey previous scholarship seeking to explain the spatial manifestation of vital inequality and articulate a unified theory of hyperincarceration as a vector of ill health. Our argument centers around the dual nature, both material and symbolic, of human capabilities to function and flourish, upon which the punitive regulation of social ills is hypothesized to have a durably corrosive impact. Second, we present a multistage empirical analysis of county-level life expectancy and premature mortality risk in which we compare and contrast the explanatory power of various variables of social scientific interest and where we also introduce a compound instrumental variable for incarceration suited to isolating exogenous treatment variation within counties over time. We complement our panel models with an investigation of between-county inequalities using a matched regression approach. Finally, we discuss the implications of our analysis for population health and the future of public policy. Our findings provide new empirical evidence surrounding the nexus of punishment and population health, while also introducing a compound instrumentation technique that can readily be employed by other scholars of incarceration.

## What Explains Spatial Variation in Population Health?

In social scientific approaches to the study of population health, vital inequalities are usually explained with reference to fundamental social causes^4^—including cultural frames^5^ and institutional arrangements^6^—rather than biological fixity or lifestyle^7^. Although behavioral factors such as diet and smoking are acknowledged as important, they are viewed as being located downstream on the causal chain leading to poor health and premature death. A distinctively social scientific approach is to relate the distributional dynamics of population health to the wider determinants of unequal life chances. These determinants, however, are typically used to account for disparities in individual-level outcomes rather than broader spatial configurations. The most dominant framework for studying key outcomes at a higher level of aggregation is offered by the rich literature on neighborhood effects wherein community contexts are viewed as powerful predictors of well-being,^8^ but there is—to our knowledge—no explicit theoretical account of what drives macro-level geographical heterogeneity in population health in the United States (or elsewhere). The textbook answer to the question “what accounts for regional disparities in life expectancy?” would most likely be fourfold: material deprivation, race, crime and violence, and access to health care. Key explanatory variables would thus include income and poverty, labor market conditions and employment status, ethno-racial and demographic factors, as well as health insurance coverage.

In this article, we empirically test a series of interrelated hypotheses corresponding to this theoretical approach—that geographical variation in material, demographic, and institutional conditions drives geographical variation in population health outcomes. To the default list of hypothesized explanatory variables, we add an important object of social scientific inquiry that forms a distinctive feature of American public policy, namely, high rates of incarceration. Not only have social scientists been interested in explaining gargantuan growth in the nation's correctional population—which has ballooned to reach more than 2 million individuals behind bars and almost 7 million individuals in total^9^—but they have also examined how this punitive upsurge has impacted durable patterns of social inequality.^10^ In particular, the criminal justice system has been construed as a vector of unequal life chances, in the broadly Weberian sense of probabilistically defined opportunity structures by which groups and individuals access and appropriate social goods. However, the precise nature of the relationship between high rates of incarceration and human welfare remains unclear, as previous scholarship has tended (more often than not) to ignore that form of inequality of which the notion of “life chances” offers an almost literal echo, namely inequalities in life and death. In the following section, we provide an empirical and theoretical rationale for our emphasis on the penal state.

## Punishment and Population Health: Theory and Evidence

In this article, we draw on a growing body of scholarship that understands population health not only as socially mediated but also as a locus of distributional conflict.^3^ According to this line of argument, human organisms are embedded within social constellations that are structured by the institutional organization of social power.^11^ Vital inequalities are therefore rooted in the durably institutionalized power of some over the material life chances of others. We take our cue from this literature by viewing the prison as a potent force in the (re)making of vital inequality insofar as it molds, from above, the social relations that impinge on human welfare: it is a politically rooted institution that actively stratifies a population, delineates and aligns group boundaries, and thus “realizes”^12^ material and symbolic divisions.^13^

We consider high rates of incarceration—or, more precisely, hyperincarceration^14^—to form part of a broader public policy repertoire that, in turn, is expressive of distributional struggles. This conceptualization of penality as public policy is historically motivated, as the initial conception of the prison in late 16th century Europe was as “an instrument of social policy with regard to beggars”^15^ geared toward the coercive regulation of poverty at the dawn of modern capitalism. A means of warehousing landless vagrants uprooted by the enclosure movement and of curbing the social convulsions wrought by the sudden commodification of labor, the carceral wing of the state was thus, from its origins, an institutional force capable of conferring upon the social determinants of health their causal efficacy. Contemporary evidence has shown that America's punitive upsurge constitutes a similarly oriented way of managing various social ills—from economic deprivation to ethno-racial enmity—via the ideology of “law and order.”^9,14,16^ However, due to the politics of federalism and locally nested differences in organizational cultures that shape the political use of public and private institutions, there is regional variation in the kinds of policy scripts that prevail in each location. Such a structural variation can help account for geographically patterned mortality burdens across the United States.^17^ This is what motivates our hypothesis of a spatially manifested linkage between incarceration and vital inequality.

## Causes and Mechanisms

From a rich body of prior research,^18,19^ we identify 3 principal mechanisms by which punitive social policies, as epitomized by high rates of incarceration, can impact human well-being. *Relegation* is the mechanism by which the criminal justice system causes groups and individuals to experience downward social mobility. This happens not only through immediate physical seclusion but also, subsequently, through the indelible blemish of a criminal record,^20^ which increases the likelihood of experiencing poverty^21^ and cements a sense of social dishonor.^22,23^ Relegation impacts not only those who are incarcerated but also the communities in which they live, where the removal primarily of working-age men has permanent spillovers in the form of disrupted social ties and territorial stigmatization. Social scientists have paid particular attention to how parental incarceration impacts “fragile families”^24^ and their neighborhoods,^25–27^ precipitating a sequence of intergenerationally transmitted and spatially concentrated adversities that shape functional development over the life course.^28–31^ In short, relegation is a mechanism whereby incarceration acts upon and activates the social determinants of health.

*Amplification* is the mechanism by which already operant social determinants of health are magnified or exacerbated. A key example is the tangled interconnection between poverty and punishment fostered by the punitive treatment of social disadvantage. Evidence suggests that aggressive policing tends to target activities such as public urination, rough sleeping, or begging for food.^32^ At the same time, whereas rates of homelessness are 21 per 10 000 population for the general public, for those who have been imprisoned once or more than once, the corresponding numbers are 141 and 279, respectively. In other words, for those with multiple encounters with the criminal justice system, homelessness rates are 13 times that of the overall populace. According to data from the Bureau of Justice Statistics, high rates of recidivism imply that almost half of those released from prison are rearrested within 1 year, more than two-thirds within 3 years, and more than 80% within 9 years, thus creating a structural interlock between penal confinement and material hardship.^33^ Hyperincarceration, then, can work as an amplifier of the social causes of ill health among society's most vulnerable.

The third and final mechanism is that of *corrosion*, which creates and compounds various modalities of “social sundering.”^3^ This involves the enduring decline of social cohesion driven by fractured social networks, neighborhood violence, and fading collective imaginaries.^34^ The importance of stable systems of meaning and collective representation, of recognition and reciprocity, and of communal cooperation and solidarity to the fostering of welfare and well-being has been highlighted in previous social scientific scholarship on population health.^5,8^ We connect this insight to empirical research on incarceration by arguing that punitive interventionism at the bottom of the class structure inflicts long-lasting harm not only by socially relegating groups and individuals or by amplifying preexisting adversity, but also by warping the moral valence of salutogenic social relations. This is conveyed, on the one hand, by the high risk of death in the immediate period after release from prison, especially from suicide or violence,^35,36^ and, on the other hand, by impacted local communities being thrust into collective disarray.^22,25^ Such fissuring of social ties and the breakdown of moral orders are potent means by which population health is durably affected.

Running through all 3 mechanisms are 2 distinct modes of causal efficacy. On the one hand, the impact of criminal justice on population health can occur acutely, such as in the case of police killings and their spillovers^37^ or the adverse experience of parental incarceration, for parents and children alike.^30,38,39^ On the other hand, the rapid growth of the carceral state over several decades has translated into a set of chronic exposures embedded in the “wear and tear” of everyday life. It is plausible to surmise that the emergence of incarceration as a normalized stage in the life course of young African-American men with low levels of formal education^40^ forms a vector of cumulative biological burdens whereby the lived experience of social adversity is deposited in the human body in the form of neuroendocrine traits that govern core pathogenic parameters,^41–43^ including the transcriptional dynamics of the genome.^44,45^ Both acute and chronic stressors can thus be at work in mapping punitive social policy to vital inequality.^46^

In summary, we posit that hyperincarceration is a powerful institutional force that mediates, modifies, amplifies, and aggravates the dynamics of vital inequality. Our argument weds theory and evidence from prior research, yet identifies a gap that our subsequent empirical analysis seeks to address. Although previous studies have shed light on the effects of imprisonment on health at the level of individuals and communities, evidence at the population level remains sparse,^47^ especially when it comes to the assessment of premature mortality^48^ and its distinctively spatial patterning.^1,2^ In addition, most of the extant literature relies on a limited number of data sets and methods that produce causal identification strategies of varying plausibility,^18^ notably in constructing appropriate comparison groups.^49,50^ In what follows, we seek to avoid these pitfalls by using a new county-level data set and a novel instrumentation technique suited to isolating exogenous treatment variation.

## Hypotheses, Data, and Methods

### Hypotheses

Against this conceptual and empirical backdrop, our principal hypothesis is that variation in population health can, at least in part, be explained by variation in rates of incarceration, above and beyond other economic and institutional factors. Through whatever pathway—be it relegation, amplification, or corrosion—we find it plausible that areas experiencing significant expansions of their penal apparatus and rapid swelling of their correctional populations will suffer from poorer human welfare, and that this association is causal. Our secondary hypothesis is that this effect will manifest strongly across the human life course, but especially before one reaches old age. This is not only because the criminal justice system targets younger age groups, but also in light of prior evidence on the intergenerational impacts of parental incarceration on childhood and adolescence. Finally, we expect the impact of punitive social policy to be durable such that its effects are manifested in the short, medium, and long run.

### Data

We use 3 alternative outcome variables to operationalize vital inequality. The first is life expectancy at birth, one of the most common indices of human welfare. However, this measure is more sensitive to child mortality than to mortality in adult life, and may thus shroud heterogeneity across the life course. For this reason, we also examine 2 alternative measures of premature mortality risk: the probability of dying between the ages of (1) 25 and 45 years and (2) 45 and 65 years. Given the demographic profile that is disproportionately affected by the penal state, these are the age ranges on which we expect high rates of incarceration to be most impactful. All 3 outcome variables are measured between 1983 and 2014 and are drawn from the Institute for Health Metrics and Evaluation.^51^

To test our hypotheses, we employ 2 sets of previously unavailable data at the U.S. county level. On the one hand, we use cross-sectional data from 2014—the most recent year with the best data coverage—that capture key variables of social scientific interest. These are county-level median household income (in constant U.S. dollars); unemployment rates; labor force participation rates; poverty rates (as per the federal poverty line); absolute income mobility (defined as the fraction of children earning more than their parents); income inequality (measured by a Gini index within the bottom 99% of the income distribution); residential segregation by race; the population fraction of African-Americans, Hispanics, or other ethnic minorities; the population fraction of high school graduates; the percentage of the population without any form of health insurance; violent crime rates; and prison incarceration rates (see below). In addition, to capture (part of) the historical legacy of institutionalized racial domination, we hand-code a binary indicator of whether a state is a former slave state or not. These data and their sources are defined and described in Supplemental Appendix Tables A1 and A2.

On the other hand, we use county-level panel data between 1983 and 2014. Our treatment variable is the county-level annual prison admissions rate, generated by the Vera Institute of Justice using state corrections sources and the National Corrections Reporting Program by the Bureau of Justice Statistics, which are compiled into annual county-level rates per 100 000 residents aged 15 to 64 years.^52^ Six states—Alaska, Connecticut, Delaware, Hawaii, Rhode Island, and Vermont—are excluded from the analysis due to the lack of consistently collected prison admissions data. Due to certain discrepancies between our data sources in measuring county boundaries and accounting for changes to counties over time, the state of Virginia and a handful of counties from other states are also excluded from the final analysis. From the above list of variables for which multiple county-year observations are available, we employ a set of baseline controls that are associated with both the treatment and the outcome, namely, median household income, annual rates of violent crime, and the fraction of each county population that is African-American. These variables are available from the U.S. Census Bureau, except for the measure of violent crime, which is extracted from the Federal Bureau of Investigation's Uniform Crime Reporting Program. Descriptive statistics for the panel data are reported in Supplemental Appendix Table A3 and further definitions are provided in Supplemental Appendix Table A1.

### Methods

We commence our analysis with a series of simple cross-sectional regression models, estimated using ordinary least squares, in which each of the 3 outcome variables is regressed against a hypothesized explanatory variable, coupled with state fixed effects. To discern differences in explanatory power and to avoid issues of multicollinearity, we present a separate model for each predictor. To test our hypotheses regarding the driving forces of spatial heterogeneity in population health, we then compare (1) coefficient sizes and (2) relative explanatory power in terms of *R*^2^ across all models.

**Figure 1.**
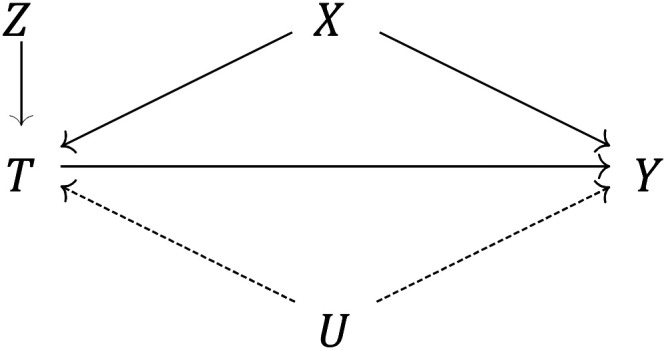
Causal graph depicting the effect of the treatment variable (*T* = incarceration) on the outcome (*Y* = life expectancy or premature mortality risk), identified via a compound instrument (*Z* = unit-specific average incarceration rate × annual nationwide correctional spending), net of both measured covariates (*X* = {household income, violent crime, demographics}) and unmeasured confounders (*U* = {eg, environmental shocks, local welfare policy}).

To examine the causal relation between incarceration and health, we posit the following data-generating process:

(1) $$Y_{it}=T_{i[t-k]}\beta +X_{it}\theta +\mu_{i}+\phi_{t}+\varepsilon_{it}$$

where *Y_it_* denotes 1 of the 3 alternative outcome variables as measured in county *i* at time *t*; the treatment variable *T_i_*_[*t*−*k*]_ is the county-level incarceration rate per 100 000 population, lagged by *k*∈{1, 5, 10} years to allow for delayed effects; *X_it_* is a vector of control variables; *µ_i_* and *ϕ_t_* capture unit and time fixed effects, respectively; and *ε_it_* is a stochastic error term. Our principal quantity of interest is *β*, which is a causal effect parameter to be estimated. However, in an observational study such as this, we do not control the source of variation in the treatment variable, nor can we know for sure if our controls are sufficient to isolate exogenous variation in the treatment. Corresponding parameter estimates may therefore suffer from endogeneity bias. We visualize this identification problem in Figure 1, where the estimated relation between the treatment variable *T* and the outcome variable *Y* may be biased by some unmeasured confounder *U*, even after controlling for observed covariates *X*. In our case, *U* might denote unobserved variables that simultaneously affect incarceration and population health, such as locally contingent environmental shocks or welfare-related policy changes.

One possible solution to this issue is to construct an instrumental variable *Z* that is correlated with the treatment but uncorrelated with any other variables in the causal system, thereby isolating exogenous variation in *T*. We propose a compound instrument derived from the interaction between the unit-specific average exposure to incarceration and the annual nationwide correctional expenditure per capita. In other words, $Z_{it}={\bar{T}}_{i}\times C_{t}$, where $\bar{T}$ is county *i*'s average incarceration rate over the sample period and *C_t_* is the aggregate per capita expenditure on the construction and maintenance of correctional facilities across all states in year *t*. The latter variable is obtained from the Bureau of Justice Statistics' Justice Expenditure and Employment Series and is measured every few years. A spline function is then applied to impute missing values through interpolation between observed years, the result of which is visualized in Supplemental Appendix Figure A1. We thus obtain a 2-stage regression model with the following selection equation:

(2) $$T_{it}=Z_{it}\tau +X_{it}\eta +\alpha_{i}+\delta_{t}+\upsilon_{it}$$

We then re-specify the model in Equation (1) as follows, with $\hat{T}$ being a vector of fitted values from Equation (2):

(3) $$Y_{it}={\hat{T}}_{i[t-k]}\beta +X_{it}\theta +\mu_{i}+\phi_{t}+\varepsilon_{it}$$

Any instrumental variable has to meet 2 principal criteria. First, the relevance criterion requires that the instrument is in fact predictive of variation in the treatment. In other words, there must exist a pathway from *Z* to *T* such that aggregate per capita correctional spending correlates with county-level rates of incarceration. To empirically assess the strength of the chosen instrument, we compare the model in Equation (2) to a restricted first-stage regression in which the effect *τ* of *Z* on *T* is set to be null, obtaining a *χ*^2^ test statistic of 2310 on 1 degree of freedom (*P <* *.*001). Hence, *Z* comfortably satisfies the benchmark for identifying a strong instrument. Second, the exclusion criterion requires that the instrument is orthogonal to the outcome over and above its effect on the treatment. The identifying assumption is therefore that the outcome of interest in counties with different propensities to incarcerate will not be affected by changes in aggregate correctional spending other than through the impact of incarceration itself: that is, as per Figure 1, the only pathway linking *Z* to *Y* goes via *T*. We thus argue that the instrument is exogenous because unit-specific shocks in incarceration that deviate from a county's long-run average are generated only by punitive policy shifts that occur independently of any particular county. We control for the endogenous relation between *T* and *Y* potentially induced by any time-invariant propensity of counties with a prior health disadvantage to adopt more punitive policy measures by adjusting for county fixed effects, whereas year fixed effects help account for broader changes in the national public health landscape that affect all counties simultaneously.

Other instrumental variable approaches in the extant literature have relied either on broader measures of aggregate-level policy environments^53^ or on individual-level variation derived from the random assignment of judges with different sentencing propensities.^54^ A recent study by Weidner and Schultz^55^ uses a cross-sectional design in which correctional spending alone is used as an instrumental variable. We believe that the methodological setup of our article provides a more stringent framework for causal inference by virtue of the time-series dimension of the data. Not only are year- and unit-specific attributes netted out by demeaning through entities, but lagged effects are also incorporated into our model design to capture short-, medium-, and long-run shocks. The 2-way fixed effects model thus constitutes a rigorous approach that eliminates any confounders that either remain stable over time—such as county- or state-level institutional factors—or form part of any aggregate time trends, while also allowing for dynamic relationships. This combination of factors leads us to believe that we are better positioned to isolate exogenous shocks that operate above and beyond individual units' default exposure to incarceration.

A central threat to our identification strategy is the presence of nonparallel trends across counties with different treatment exposure levels. This is because our approach is akin to a difference-in-difference design wherein the effect of *T* on *Y* is compared across counties assigned to (continuous) treatment states as a function of nationwide correctional spending over time. The key assumption of such a design is that the control units provide an appropriate counterfactual of the trend that the treated units would have followed had they not been treated. To test this assumption, we visually inspect aggregate trends for all 3 outcomes of interest, stratified by whether units experience above or below mean exposure to incarceration. As reported in Supplemental Appendix Figures A2 to A4, we find no indication of the parallel trends assumption being violated.

Another threat to causal inference is the persistence of residual confounding. Given that we cannot empirically verify that our instrument is strictly exogenous, the probability of such unmeasured confounding is nonzero. To address this concern, we conduct a simple nonparametric sensitivity analysis that allows us to precisely quantify the amount of unmeasured confounding that would in theory be required to eliminate our estimated treatment effect $\hat{\beta }$. Let

$$\hat{\beta }\;=E(Y|T=1,X)-E(Y|T=0,X)$$

denote the expected difference in the outcome variable *Y* for (theoretically dichotomized) treatment and control units, respectively, net of our matrix of controls *X*, and let *U* denote an unmeasured confounder. Then the bias factor, *B*, is defined as the difference between $\hat{\beta }$ and what $\hat{\beta }$ would have been had we controlled for *U* as well. We make the simplifying assumptions that *U* is binary and that the effect of *U* on *Y* is the same across both treatment states (i.e., no *U *×* T* interaction). For the most part, these assumptions merely serve to simplify the presentation without substantively impacting the outcome of the sensitivity analysis. We now define

**Figure 2.**
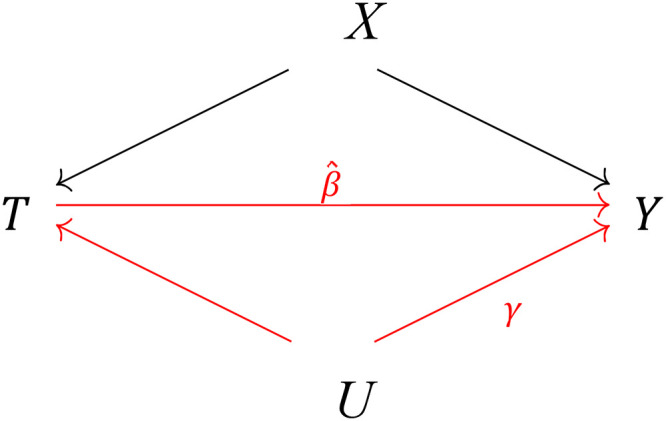
Causal graph depicting the potential sensitivity of the estimated effect $\hat{\beta }$ of the treatment variable (*T* = incarceration) on the outcome (*Y* = life expectancy or premature mortality risk) to unmeasured confounding (*U* = {eg, environmental shocks, local welfare policy}), net of a set of measured covariates (*X* = {household income, violent crime, demographics}).

γ=E(Y|U=1,T,X)−E(Y|U=0,T,X)

as the effect of the unmeasured confounder on the outcome, net of the treatment and control variables, as visualized in Figure 2. We also define

δ=P(U=1|T=1,X)−P(U=1|T=0,X)

as the difference in the prevalence of the unmeasured confounder between the treatment and control groups. The bias factor is then readily obtained as the product of these 2 sensitivity parameters: *B* = *γ* × *δ*.^56,57^ In assessing the sensitivity of our model coefficients to unmeasured confounding, we ask how large *γ* would have to be to reduce our estimated effect size $\hat{\beta }$ to 0. We address this question by visualizing how *B* changes as the 2 sensitivity parameters (co-)vary across a range of possible values.

### Assessing Between-County Variation

Although fixed effects regressions are nearly always preferred in analyses of panel data, we complement the investigation of within-county variation over time with an analysis of between-county variation. This is for 2 principal reasons. On the one hand, despite its many virtues, the “within” estimator eliminates most of the variation—and hence information—captured by the data and does not always lend itself to readily interpretable predicted values of substantive interest. On the other hand, by isolating inequalities across units, the “between” estimator is geared toward our principal quantity of interest, namely, geographical disparities. This allows us to simulate sociologically relevant counterfactuals and compare differences in outcomes in an intuitive way.

However, we acknowledge the methodological flaws of the “between” estimator—which are compounded by the fact that we cannot instrument for incarceration in a cross-sectional setting. To render the corresponding parameter estimates more plausible, we employ coarsened exact matching as a nonparametric form of preprocessing the data.^58,59^ The goal of matching is to reduce inefficiency, bias, and model dependence by selecting units of analysis that are similar to one another in all respects except for their treatment status. Thus, in our case, counties are “matched” with other counties that share key characteristics, with the exception that some have high incarceration rates and others have low incarceration rates. This will facilitate a more precise account of the link between penal expansion and vital inequality. In formal terms, we let *Y* denote the outcome variable of interest, *T_i_*∈{0, 1} is a dichotomized treatment indicator encoding below versus above mean exposure to incarceration, and *X* is the set of pretreatment covariates (violent crime, median household income, high school graduation rates, and the county ethno-racial composition). The treatment effect *β* on a treated unit *i* is *β_i_* = *Y_i_*(*T_i_* = 1) − *Y_i_*(*T_i_* = 0). However, the last term of this equation, *Y_i_*(*T_i_* = 0), is an unobserved counterfactual. One can estimate this quantity with *Y_j_* from control units (indexed by *j*) that are matched on relevant covariates (ie, *X_i_* ≈ *X_j_*) such that the estimated counterfactual quantity, $\hat{Y_{i}}(T_{i}=0)$, is equal to *Y_j_*(*T_j_* = 0). Coarsened exact matching temporarily “coarsens” the covariates *X* into subcategories using a nonparametric histogram estimator. It then applies exact matching on the coarsened *X*, *c*(*X*), before sorting observations into strata, each with unique values of *c*(*X*). Any stratum with 0 treated or control units is pruned from the data set. The algorithm then passes the original (uncoarsened) units—except for the pruned ones—on to the matched data set that is used in the parametric analysis (for further details, see the work of Iacus et al^60^). All analyses are conducted in R version 4.0.2 (R Core Team 2020).^61^

## Findings

### Preliminary Models

A set of preliminary results are displayed in Table 1. These models include key hypothesized predictors of the 3 outcome variables. To avoid issues of multicollinearity, only a subset of available variables are selected on the basis of overall model fit. In particular, the variable measuring the population percentage without health insurance turns out to be highly correlated with median household income and is therefore omitted from these specifications. Including the variable does not alter any other parameter estimates, but reverses the sign of the variable itself whenever it is used together with a median household income. We do, however, assess the robustness of our principal findings to controlling for health insurance coverage (and other covariates) in our sensitivity analysis (see below). Moreover, all models are adjusted for state fixed effects. Each outcome variable is log-transformed, while all continuous predictors are standardized by subtracting the mean and dividing by the standard deviation. Parameter estimates are thus interpreted as the (approximate) percentage change in the outcome variable associated with each standard deviation increase in the predictor. We find, as suspected, that local economic conditions, in the form of median household income and unemployment rates, are strongly associated with cross-county variation in life expectancy and premature mortality risk. The same goes for regional variation in the ethno-racial composition of counties, as well as variation in violent crime rates. In an alternative set of model specifications (not displayed), we replace the state fixed effects with an indicator of whether or not a county is located within a former slave state. This is associated with percentage changes in each of the 3 outcome variables by −1.0% (95% confidence interval [CI]: −1.2, −0.8; *P* *<* *.*001), 11.5% (95% CI: 9.7, 13.3; *P* < *.*001), and 7.9% (95% CI: 6.4, 9.5; *P* *<* *.*001), respectively. Overall, these findings suggest that the social scientific inclination to look for economic and historical institutional explanations of geographical heterogeneity in population health is well founded. Moreover, we find that our emphasis on the penal state is justified: higher rates of incarceration are significantly associated with all 3 outcomes above and beyond the other covariates, and they contribute meaningfully to the overall model fit. However, these simple correlational findings do not allow us to draw any strong conclusions, especially when it comes to questions of causality. To assess the causal nature of the observed association between incarceration and population health, we proceed to our instrumented panel data analysis.

**Table 1.** Preliminary Cross-Sectional Regression Models.

	Life expectancy	*P* (death 25-45)	*P* (death 45-65)
Median household income	1.0%	−12.0%	−9.4%
	(.1)	(.6)	(.4)
Unemployment rate	−0.6%	5.4%	3.2%
	(.1)	(1.0)	(.7)
Fraction African-Americans	−0.2%	1.8%	1.6%
	(.1)	(.6)	(.5)
Violent crime rate	−0.2%	2.4%	2.3%
	(.05)	(.4)	(.4)
Prison incarceration rate	−0.5%	3.7%	4.0%
	(.1)	(.5)	(.4)
*R* ^2^	76%	81%	79%
Observations	1990	1990	1990

*Note.* The log-transformed outcome variables are life expectancy at birth, the risk of death between the ages of 25 and 45 years, and the risk of death between the ages of 45 and 65 years. All models are adjusted for state fixed effects. All regressors are standardized by subtracting the variable mean and dividing by its standard deviation. Parameter estimates are interpreted as the percentage change in the outcome variable associated with a standard deviation increase in each predictor. Robust standard errors are shown in parentheses. All parameter estimates are statistically significant at *P *< *.*001.

### Panel Regressions

Table 2 displays results from 3 separate 2-way fixed effects instrumental variable regressions in which, once again, the outcome variable is log-transformed and the treatment variable is standardized by subtracting the mean and dividing by its standard deviation. This allows us to interpret the treatment effect as the percentage change in the outcome caused by a standard deviation increase in rates of incarceration. The variables are also residualized with respect to violent crime rates, median household income, and the fraction of the county population who are African-Americans, such that coefficients are interpreted as net effects. All variance estimators are consistent with serial autocorrelation, heteroskedasticity, and unit clustering. We observe that a standard deviation increase in the treatment variable in 1 year is estimated to cause a 0.7% drop in life expectancy at birth in the following year (95% CI: 0.5, 0.8; *P *< *.*001). The short-term risk of dying between the ages of 25 and 45 years is increased by 7.8% (95% CI: 6.6, 8.9; *P *< *.*001), whereas the risk of death for the 45 to 65 age group is increased by 8.1% (95% CI: 6.9, 9.2; *P*< *.*001). These are substantively large effect sizes that offer strong support in favor of our principal hypothesis, namely, that punitive social policy, operationalized as high rates of incarceration, has a detrimental impact on human welfare. Moreover, the expectation that health in midlife is most heavily affected appears to be vindicated. Finally, we confirm that penal expansion has substantively large medium- and long-term consequences for population health, as evidenced by robust treatment effects after 5 and 10 years.

**Table 2.** 2-Way Fixed Effects Instrumental Variable Regression Models.

	Life expectancy	*P* (death 25-45)	*P* (death 45-65)
Incarceration rate (*t* −* *1)	−0.7%	7.8%	8.1%
	(.07)	(.6)	(.6)
Incarceration rate (*t *−* *5)	−0.5%	6.5%	7.0%
	(.05)	(.5)	(.5)
Incarceration rate (*t *−* *10)	−0.6%	6.3%	7.0%
	(.06)	(.6)	(.6)

*Note*. The log-transformed outcome variables are life expectancy at birth in the first column, the probability of death between the ages of 25 and 45 years in the second column, and the probability of death between the ages of 45 and 65 years in the third column. The incarceration variable, lagged by 1 year, is instrumented as described in the “Hypotheses, Data, and Methods” section. The corresponding parameter estimates are interpreted as the percentage change in the outcome variable caused by a standard deviation increase in incarceration rates after adjusting for violent crime rates, median household income, and fraction African-Americans. Standard errors consistent with serial autocorrelation, heteroskedasticity, and unit clustering are shown in parentheses below each parameter estimate. All parameter estimates are statistically significant at *P* < .001.

**Figure 3.**
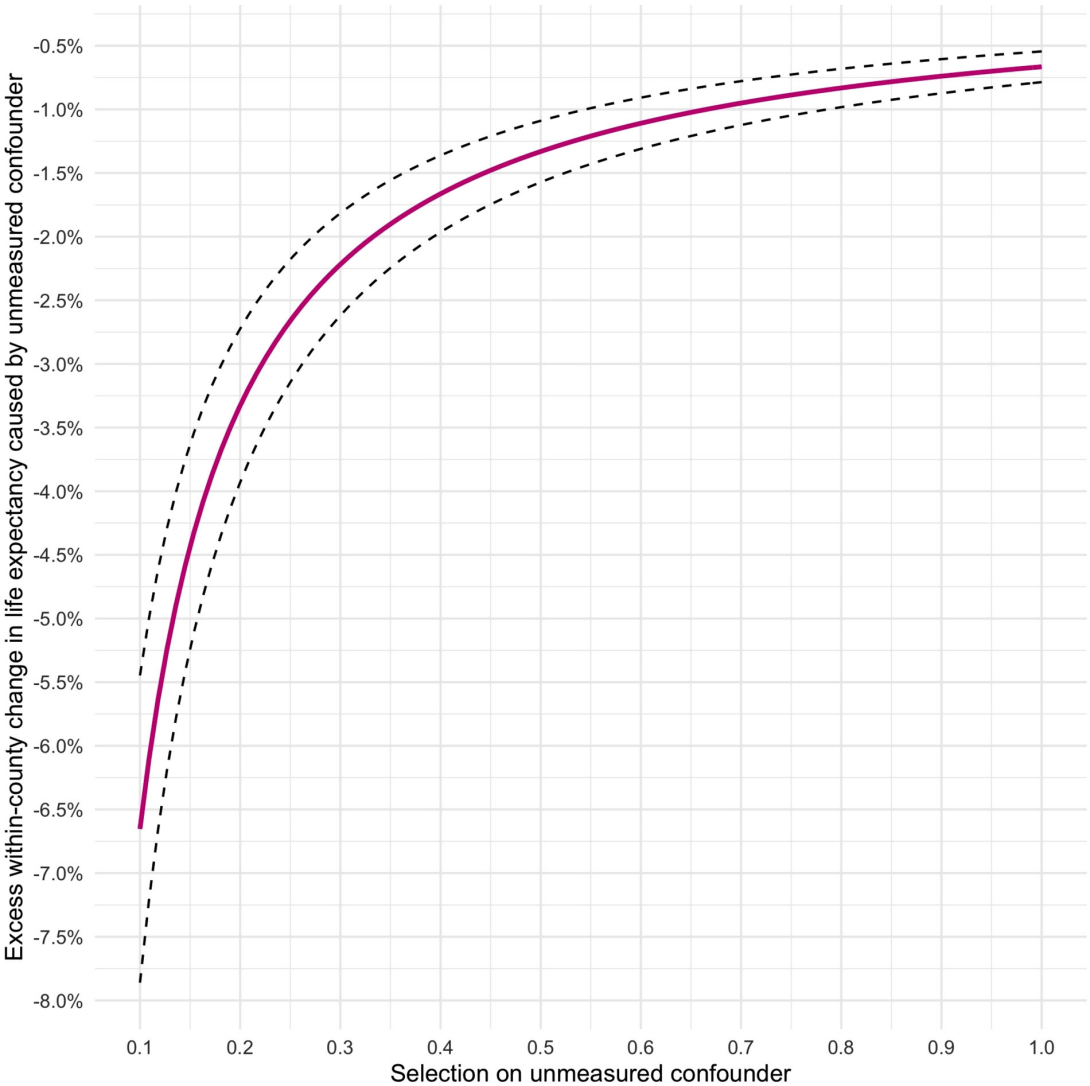
Sensitivity analysis plot to assess residual confounding of the estimated effect $\hat{\beta }$ of incarceration on life expectancy at birth as per Table 2. Values of *δ* (*X*-axis) and *γ* (*Y*-axis) that lie on the solid violet line would completely eliminate the estimated effect. The dotted black lines denote the lower and upper bounds of the corresponding 95% confidence interval. Values below the plotted curve would reverse the sign of the estimated effect.

We now assess the robustness of our estimated treatment effect to the presence of unmeasured confounding. To save space, we limit the sensitivity analysis to the first model of life expectancy, but similar (and even more robust) results apply to the other 2 outcome measures. Figure 3 visualizes variation in the bias factor *B*, as defined earlier, across a range of possible values of the 2 sensitivity parameters *δ* and *γ*. The *X*-axis denotes the degree of selection on the unmeasured confounder across the 2 treatment states (ranging from 0 to 1, with higher values indicating a higher prevalence of the confounder in the treatment group, ie, in counties with higher rates of incarceration), whereas the *Y*-axis denotes the magnitude of the effect of *U* on the outcome, above and beyond that of the treatment and the control variables, that would be required to completely eliminate the effect of incarceration on life expectancy at birth. In light of the argument concerning the exogeneity of our chosen instrument, we believe it is plausible that the amount of residual confounding—if there is any—remains moderate. As such, the most likely values of *δ* would be at the lower end of the *X*-axis in Figure 3. At, say, *δ* = 0*.*1, *U* would have to cause an excess within-county fall in life expectancy such that *γ* ≈ −6*.*5% to nullify the effect of incarceration. Given the relative magnitudes at stake, this seems highly unlikely. For the sake of argument, assume that the bias factor is 10% of $\hat{\beta }$ (i.e., *B* = 0*.*07%). Then the bias-adjusted effect of high incarceration rates would still be −0*.*63% (95% CI: −0*.*53, −0*.*73; *P *< *.*001), which is equivalent to around half a year of life expectancy. Overall, the sensitivity analysis suggests that an unusual amount of unmeasured confounding would be needed to cast doubt upon our causal estimates.

### Cross-Sectional Regressions

We proceed to preprocessing the data using coarsened exact matching. We first assign units to treatment and control groups, defined as above versus below mean exposure to incarceration, respectively. We then apply the matching algorithm, which results in a pruned data set composed of *N* = 1679 counties, with 1064 counties in the control group and 615 counties in the treatment group. Counties are matched on time-averaged versions of the covariates from the panel regression—i.e., violent crime rates, median household income, and the county fraction of African-Americans. To shore up additional between-unit heterogeneity, we also match on the county fraction of non-White Hispanics or other ethnic minority and county-level high school graduation rates. The diagnostics reveal a high degree of balance improvement since the empirical covariate distributions in both the treatment and control groups are now similar, meaning the smaller sample size strengthens rather than undermines the subsequent statistical inference. Complete matching results are reported in Supplemental Appendix Table A4. Using simple ordinary least squares, we then estimate a “between” model

$$Y_{i}=T_{i}\beta +\zeta_{i}$$

where the (time-averaged) outcome variable *Y* is regressed on *T* alone since covariate balance is obtained through matching. We log-transform *Y* and rescale *T* as done above. Table 3 displays the results of 3 separate regressions, 1 for each outcome variable. We see that a standard deviation increase in incarceration is associated with a 1.1% decline in life expectancy at birth (95% CI: 1.0, 1.2; *P *< *.*001) and a rise in the 2 measures of premature mortality risk of 10.6% (95% CI: 9.6, 11.6; *P *< *.*001) and 7.9% (95% CI: 7.2, 8.8; *P *< *.*001), respectively. We note, moreover, that incarceration appears to account for no less than one-fifth of all variation in all 3 outcome variables. For the sake of comparison, we also match counties on their incarceration rates (in addition to the aforementioned covariates) and assess the treatment effects of median household income (models not displayed). We find that a standard deviation increase in income is associated with a 1.1% (95% CI: 1.0, 1.2; *P *< *.*001) increase in life expectancy at birth, 14.4% (95% CI: 13.4, 15.5; *P *< *.*001) decline in the risk of death between the ages of 25 and 45 years, and a 9.7% (95% CI: 8.8, 10.6; *P *< *.*001) decline in the risk of death between the ages of 45 and 65 years.

**Table 3.** Between-County Matched Regression Models.

	Life expectancy	*P* (death 25-45)	*P* (death 45-65)
Incarceration rate	−1.1%	10.6%	7.9%
	(.1)	(.5)	(.4)
*R* ^2^	18%	21%	20%
Observations	1679	1679	1679

*Note*. The log-transformed outcome variables are life expectancy at birth in the second column, the probability of death between the ages of 25 and 45 years in the third column, and the probability of death between the ages of 45 and 65 years in the fourth column. The association between treatment and outcome is estimated by applying a simple linear regression model to a pruned data set that is preprocessed using coarsened exact matching. Counties are matched on the variables listed in the “Hypotheses, Data, and Methods” section (see also Supplemental Appendix Table A4). All variables are time-averaged over the sample period. Parameter estimates are interpreted as the percentage change in the outcome variable associated with a standard deviation increase in incarceration rates. Standard errors are shown in parentheses below each parameter estimate. All parameter estimates are statistically significant at *P *< *.*001.

**Figure 4.**
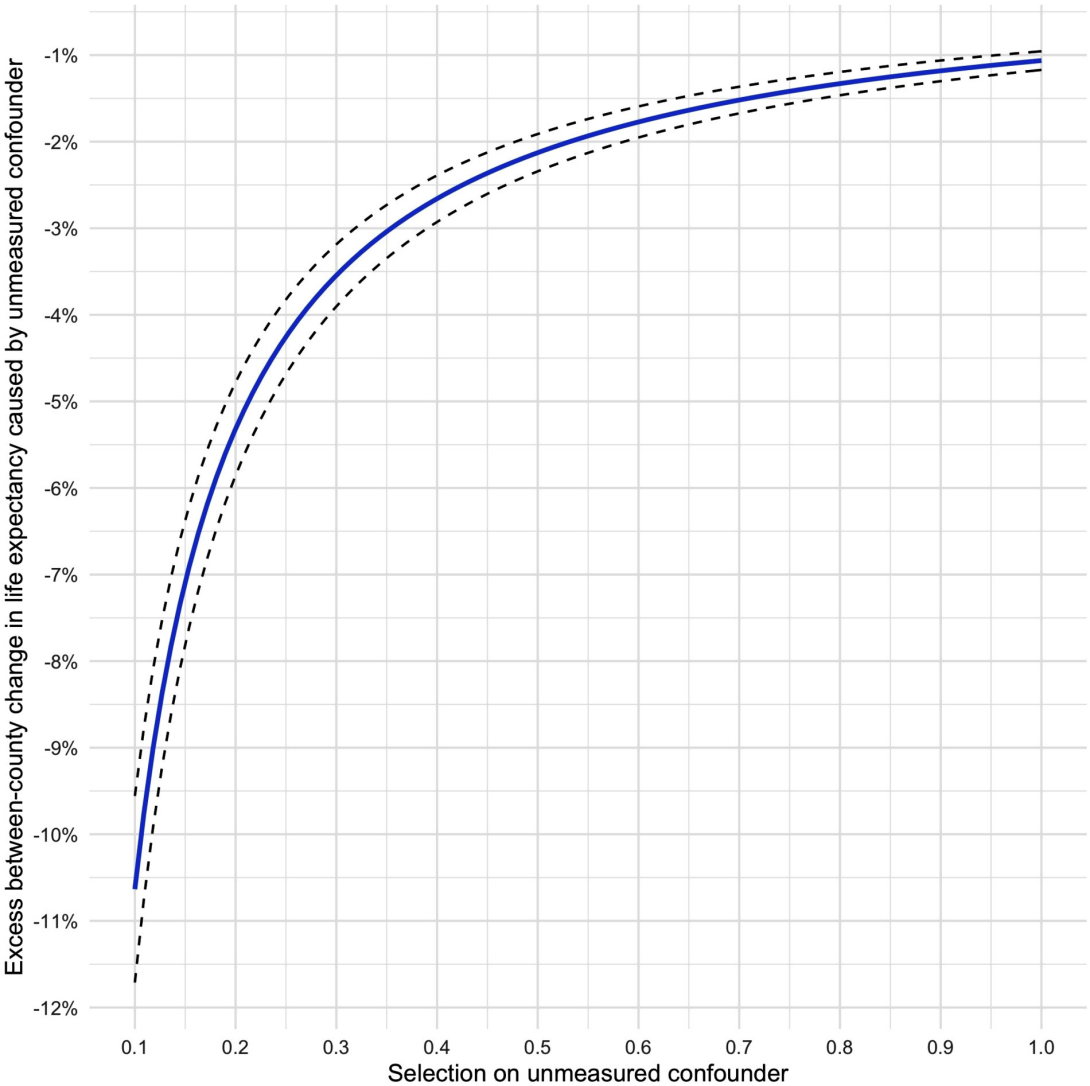
Sensitivity analysis plot to assess unmeasured confounding of the estimated effect $\hat{\beta }$ of incarceration on life expectancy at birth as per Table 3. Values of *δ* (*X*-axis) and *γ* (*Y*-axis) that lie on the solid blue line would completely eliminate the estimated effect. The dotted black lines denote the lower and upper bounds of the corresponding 95% confidence interval. Values above the plotted curve would reverse the sign of the estimated effect.

We conduct the same sensitivity analysis as before, again for life expectancy at birth, as shown in Figure 4. Given the lack of instrumentation, it is hard to surmise the amount of unmeasured confounding that is reasonable to expect in the case of this model. Nonetheless, we see that even at unusually high levels of selection on the unmeasured confounder—i.e., *δ* = 0*.*8—*γ* would still need to exceed the estimated effect of *T* to cancel out $\hat{\beta }$. At more moderate levels of *δ*, say, *δ* = 0*.*2, the net impact of *U* (or *U*) on *Y* would have to be nearly −5*.*5% to nullify $\hat{\beta }$. Overall, this indicates that a nonnegligible portion of the reported association is likely to be causal and thus that incarceration is a determinant not only of within-county changes in population health but also of between-county inequalities.

**Figure 5.**
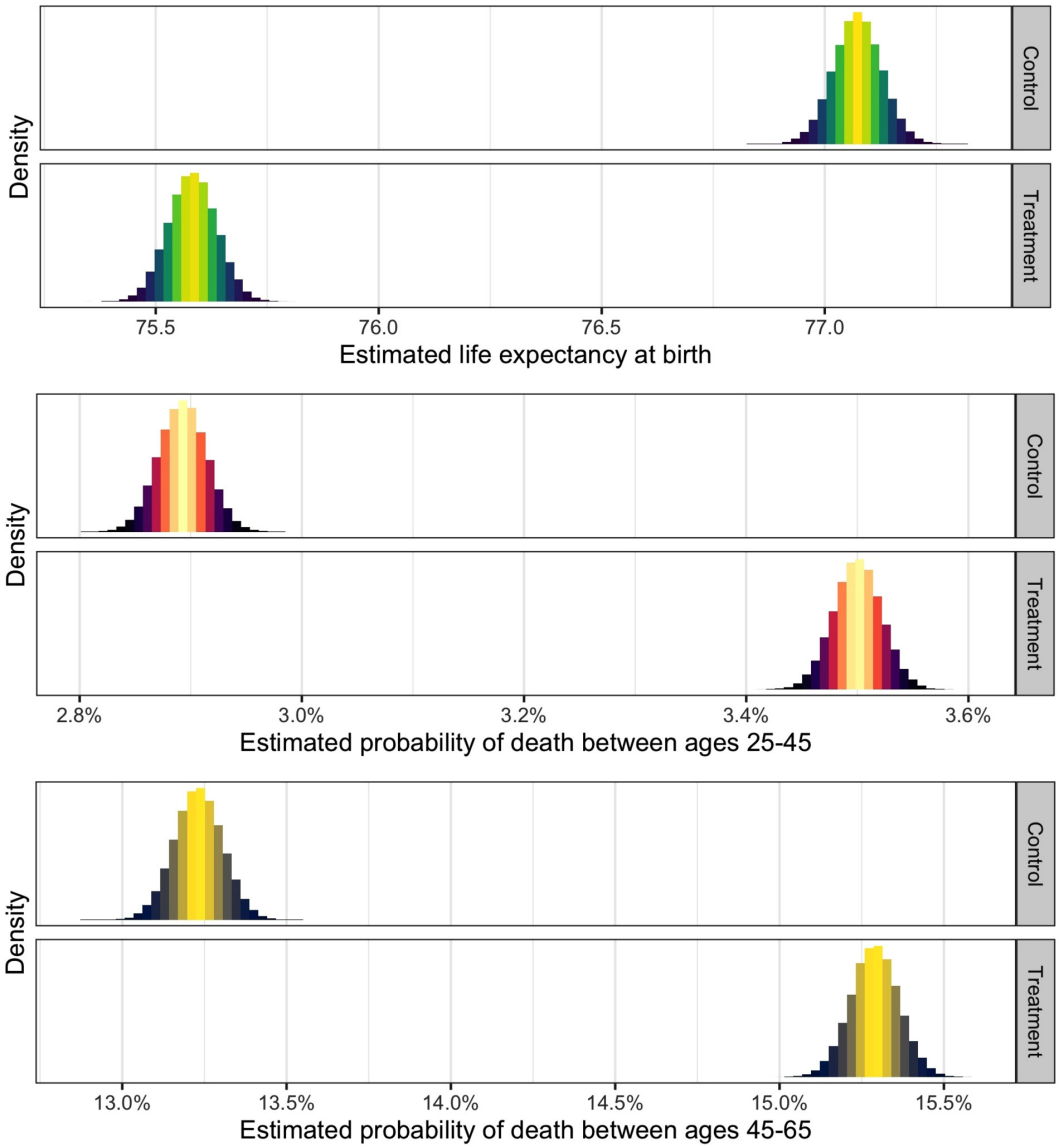
Density plots of expected outcome values conditional on treatment state. In the top panel, the outcome variable is life expectancy at birth, in the middle panel, the outcome variable is the probability of death between ages of 25 and 45 years, and in the bottom panel, the outcome variable is the probability of death between ages of 45 and 65 years. Each model compares counties with incarceration rates at 1 standard deviation below the mean (control) to those with incarceration rates at one standard deviation above the mean (treatment). The association between treatment and outcome is estimated by applying a simple linear regression model to a pruned data set that is preprocessed using coarsened exact matching. Counties are matched on the variables listed in the “Hypotheses, Data, and Methods” section (see also Supplemental Appendix Table A4). All variables are time-averaged over the sample period. *N* = 1679.

To get a better intuitive sense of what these numbers mean in substantive terms, we predict the conditional expectation of each outcome variable given different levels of *T*. We first (re)define control units as those with incarceration rates at 1 standard deviation below the mean and treatment units as those with incarceration rates at 1 standard deviation above the mean. We then calculate the corresponding conditional expectations of *Y* across 100 000 simulated values of the stacked column vector $\hat{\psi }=\{\hat{\beta },{\hat{\sigma }}^{2}\}$, derived from the outputs of a model similar to that in Table 3, except the outcome variable is no longer log-transformed and the treatment is not rescaled. This vector forms the mean of a multivariate normal distribution with variance equal to the model covariance matrix $\hat{V}(\hat{\psi })$. We may thus obtain simulated parameter values $\tilde{\psi }∼N(\hat{\psi },\hat{V}(\hat{\psi }))$, which are used to predict and plot life expectancy and premature mortality risk by treatment status, while simultaneously accounting for estimation uncertainty.^62^ The results of this procedure are shown in Figure 5. We see that a shift from low to high incarceration rates is associated with a drop in life expectancy from over 77 years in the control group to just over 75.5 years in the treatment group. When it comes to the probability of dying between the ages of 25 and 45 years, we see that high rates of incarceration shift this probability from around 2.9% in the control group to around 3.5% in the treatment group. Finally, for the 45 to 65 age bracket, a shift from low to high incarceration rates is associated with a rise in the probability of death from under 13.5% to nearly 15.5%. Overall, these results suggest that differences in incarceration rates can account for a substantial portion of disparities in health outcomes across U.S. counties.

### Alternative Specifications

For the sake of comparison and completeness, we also run a series of cross-sectional models in which we assess the robustness of the association between incarceration and health to additional control variables from 2014, as described in Supplemental Appendix Table A2. To avoid overspecification, we add and remove 1 control variable at a time. However, as above, we adjust for state fixed effects in all models. Results are displayed in Supplemental Appendix Tables A5 to A7 for each of our 3 outcome variables. We note that the estimated coefficient of incarceration remains remarkably stable across all specifications, which further confirms the robustness of the hypothesized relation between rates of imprisonment and population health.

## Concluding Discussion

Our analysis provides a unified account of substantial geographical variation in life expectancy at birth and premature mortality risk across the United States. We draw on a previous scholarship on the distal determinants of vital inequality and generate a framework for the study of population-level (as compared to individual-level) health outcomes. We relate the distributional dynamics of mortality and life expectancy to a range of sociological factors, and we spotlight 1 of these—punitive social policy—that is rarely invoked in the existing population health literature, despite being a major object of social scientific inquiry. Our findings suggest not only that rates of incarceration are *relevant* to population health outcomes, but that they exert large and enduring effects on human life chances that compete in magnitude with other major “fundamental” social causes.

We see our article as a contribution to the science of vital inequality. We provide an empirically grounded yet theoretically informed account of the causal linkages between high imprisonment rates and 3 alternative health outcomes, and our estimated effects prove to be substantively large and significant. We offer an analytically unified theory of hyperincarceration as a vector of ill health that spotlights downward social mobility, cumulative disadvantage, and the breakdown of collective efficacy as key components, and we generate causal evidence in support of our principal hypotheses using a stringent 2-way fixed effects instrumental variable regression, complemented by various investigations of between-county associations. Our findings provide new empirical insights surrounding the nexus of punishment and population health that may inform future policymaking geared toward criminal justice reform. Insofar as the prison embodies a distinctly American nexus of class and race, our analysis also speaks to ongoing political debates surrounding issues of social inequality, racial justice, and human welfare. In the process, we have introduced a novel instrumentation technique that can readily be employed by other scholars of incarceration and health.

We acknowledge the limitations of our approach. First of all, despite our systematic conceptualization of the causal pathways leading from incarceration to vital inequality, we are unable to explicitly test the workings of such mechanisms. Instead, we draw on prior literature to motivate the broader scope of our analysis. Future work should seek to probe the mechanisms of relegation, amplification, and corrosion through the use of large-scale multilevel data sources that follow individuals, neighborhoods, and broader collectives over extended periods of time. Moreover, our data, despite being of high quality and collected at a relatively fine-grained level of geographical resolution, prevent us from further disaggregating the estimated effects and examining their likely heterogeneity. We also acknowledge that our identification strategy may suffer from unmeasured confounding—especially in our account of between-county inequalities—given that we cannot be certain to have captured purely exogenous treatment variation. However, we provide a simple yet informative sensitivity analysis suggesting that an inordinate amount of confounding must be present to nullify our main results. Our models are highly robust to alternative specifications and provide substantively meaningful estimates of the hypothesized relationships. Our overall conclusion thus remains unambiguous: punitive social policy kills.

## Supplemental Material

sj-docx-1-joh-10.1177_00207314211024895 - Supplemental material for Punitive Social Policy and Vital Inequality Click here for additional data file. Supplemental material, sj-docx-1-joh-10.1177_00207314211024895 for Punitive Social Policy and Vital Inequality by Elias Nosrati and Lawrence P. King in International Journal of Health Services
