# Supplementary material for: Punitive Social Policy and Vital Inequality
Source: Int J Health Serv. 2021 Jun 9;51(4):545–58. doi: 10.1177/00207314211024895 (PMC8435832; doi:10.1177/00207314211024895)
Supplement: sj-docx-1-joh-10.1177_00207314211024895 - Supplemental material for Punitive Social Policy and Vital Inequality [file sj-docx-1-joh-10.1177_00207314211024895.docx]

# **Appendix**

Figure A1: Observed and imputed values of annual aggregate per capita correctional expenditure.

Figure A2: Visual inspection of parallel trends assumption for counties with above versus below average exposure to incarceration. Outcome: life expectancy at birth.

Figure A3: Visual inspection of parallel trends assumption for counties with above versus below average exposure to incarceration. Outcome: probability of death between ages 25–45.

Figure A4: Visual inspection of parallel trends assumption for counties with above versus below average exposure to incarceration. Outcome: probability of death between ages 45–65.

Table A1: Variable names, definitions, and sources.

Table A2: Descriptive statistics: cross-sectional data from 2014.

Table A3: Descriptive statistics: panel data from 1983–2014.

Table A4: Mean covariate balance obtained from coarsened exact matching.

Table A5: Cross-sectional regression model of life expectancy at birth.

Table A6: Cross-sectional regression model of premature mortality risk ages 25–45.

Table A7: Cross-sectional regression model of premature mortality risk ages 45–65.


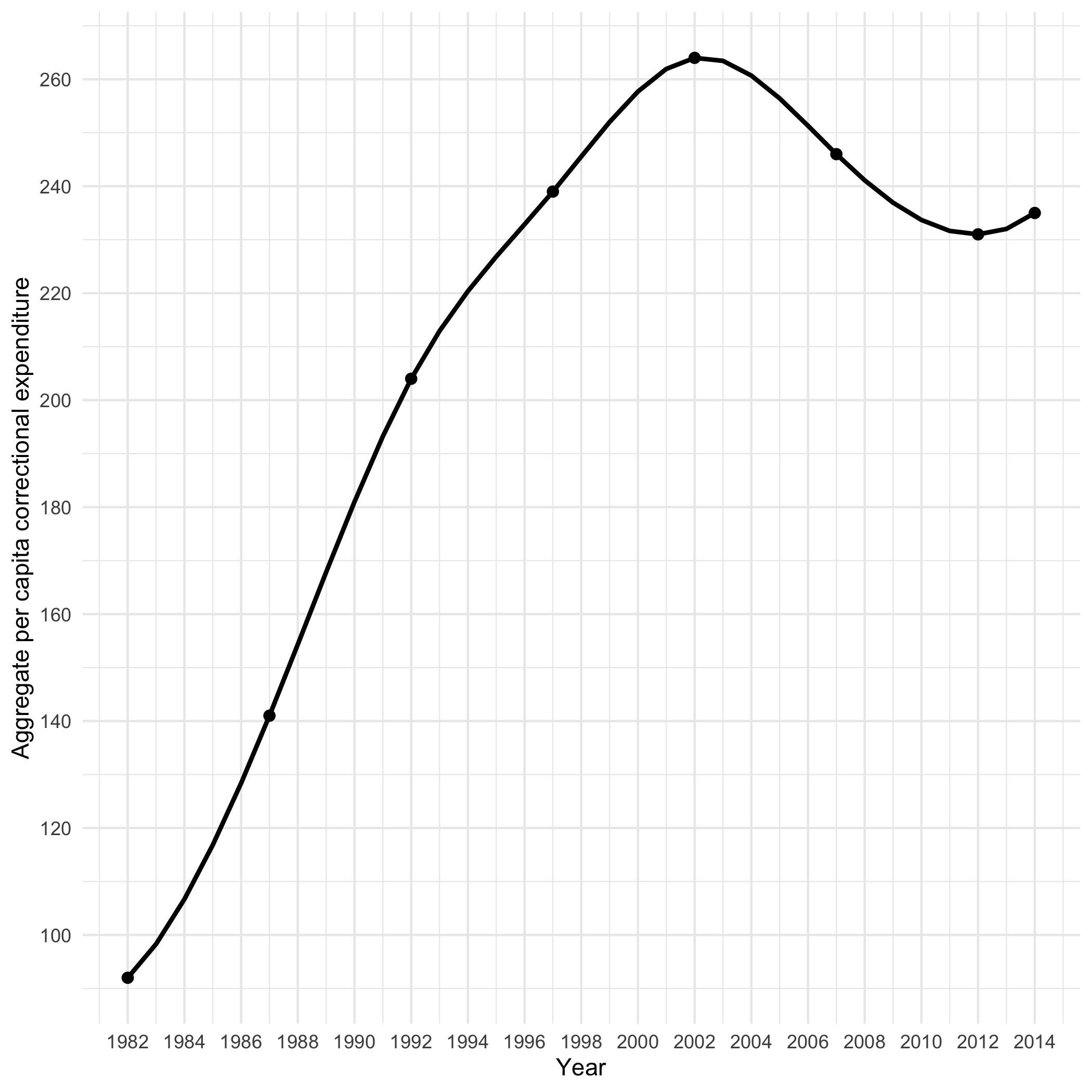


Figure A1. Observed and imputed values of annual aggregate per capita correctional expenditure.


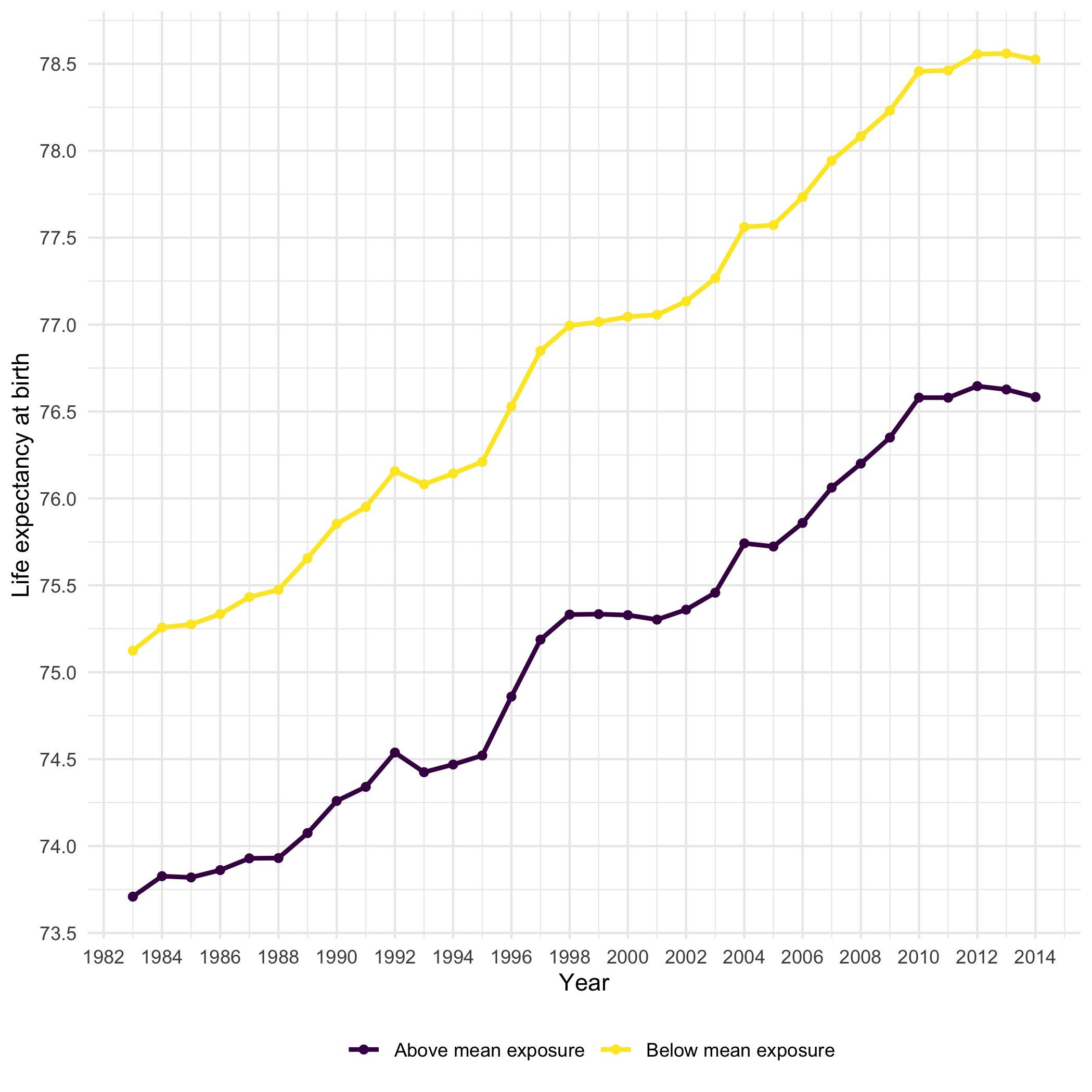


Figure A2. Visual inspection of parallel trends assumption for counties with above versus below average exposure to incarceration. Outcome: life expectancy at birth.


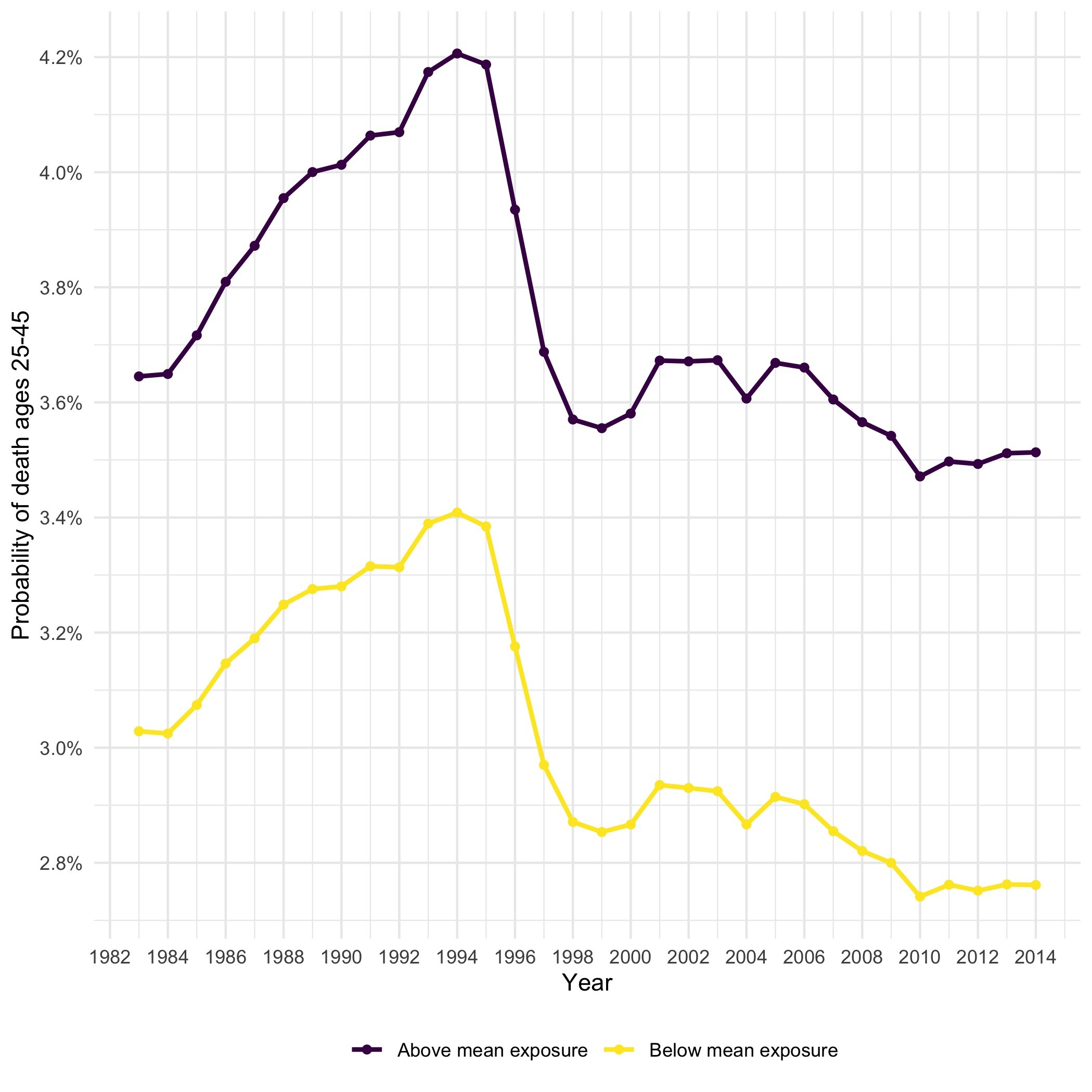


Figure A3. Visual inspection of parallel trends assumption for counties with above versus below average exposure to incarceration. Outcome: probability of death between ages 25–45.


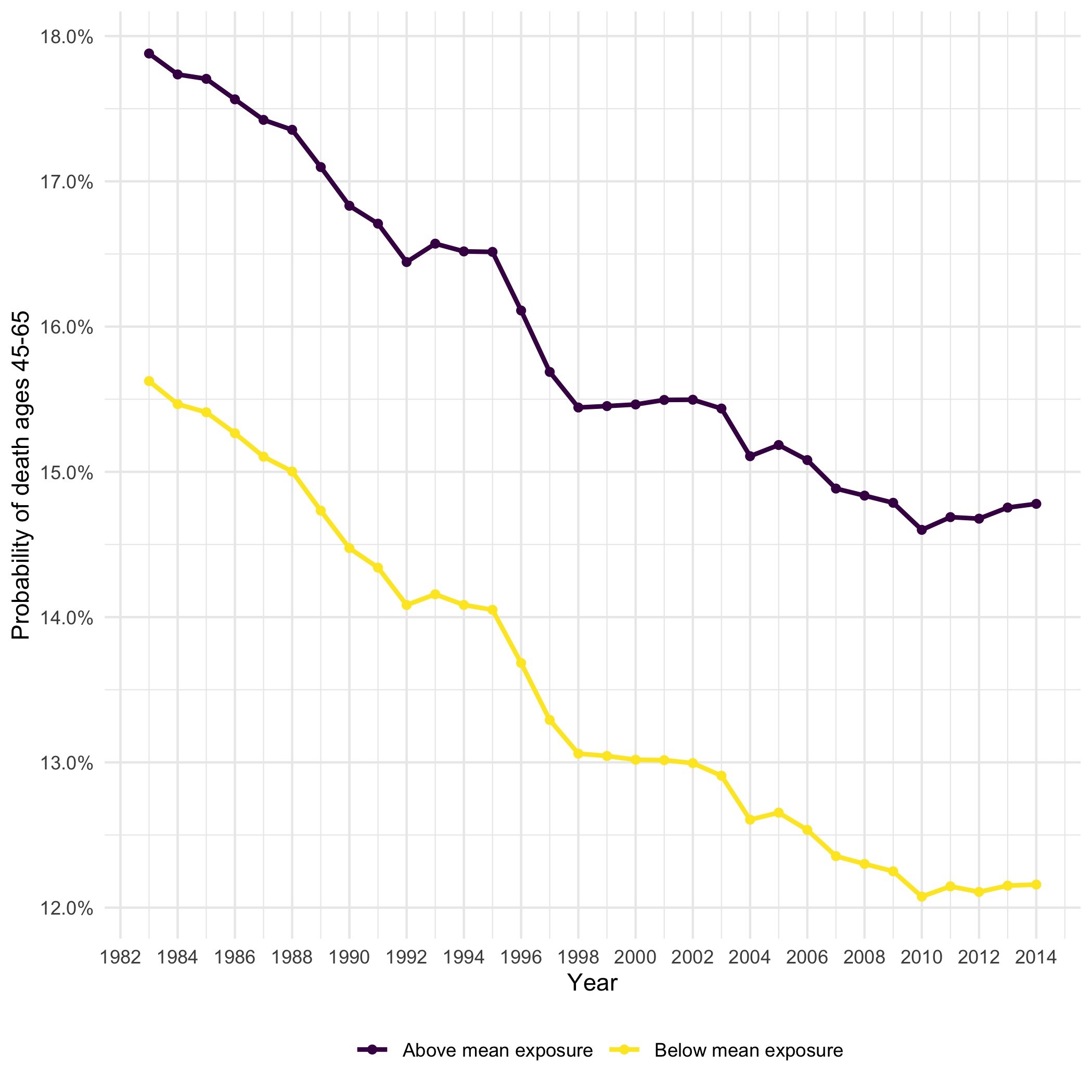


Figure A4. Visual inspection of parallel trends assumption for counties with above versus below average exposure to incarceration. Outcome: probability of death between ages 45–65.

Table A1

Variable names, definitions, and sources

**Variable Definition Source**

| Life expectancy at birth | Annual life expectancy at birth between 1983 and 2014 | U.S. National Vital Statistics System via Institute for Health Metrics and Evaluation |
| --- | --- | --- |
| Probability of death ages 25–45 | County-level annual probability of premature death between the ages of 25 and 45 between 1983 and 2014 | U.S. National Vital Statistics System via Institute for Health Metrics and Evaluation |
| Probability of death ages 45–65 | County-level annual probability of premature death between the ages of 45 and 65 between 1983 and 2014 | U.S. National Vital Statistics System via Institute for Health Metrics and Evaluation |
| Prison incarceration rate | Admissions rate to state prisons per 100,000 county population aged 15– 64 between 1983 and 2014 | Vera Institute of Justice |
| Violent crime rate | Rate of violent crime per 100,000 county population between 2006 and 2014 | U.S. Census Bureau |
| Median household income | Median county household income, measured in constant U.S. dollars, between 1983 and 2014 | U.S. Census Bureau |
| Fraction African-Americans | Fraction of county population who are African-American between 1983 and 2014 | U.S. Census Bureau |
| Fraction Hispanics | Fraction of county population who are Hispanic between 1983 and 2014 | U.S. Census Bureau |
| Fraction other ethnic minority | Fraction of county population who are other ethnic minority between 1983 and 2014 | U.S. Census Bureau |
| High school graduation rate | Fraction of county population with a high school diploma between 1983 and 2014 | U.S. Census Bureau |
| Unemployment rate | Unemployment rate per active county population in 2014 | U.S. Bureau of Labor Statistics |
| Labor force participation rate | County-level labor force participation rate in 2014 | Opportunity Insights |
| Poverty rate | County-level rate of poverty, as per federal poverty line, in 2014 | Opportunity Insights |
| Absolute income mobility | County-level fraction of children who earn more than their parents in 2014 | Opportunity Insights |
| Racial segregation | County-level spatial segregation by race in 2014 | Opportunity Insights |
| Percentage uninsured | Percentage of county population without health insurance | Opportunity Insights |
| Former slave state | Indicator of whether a county is located in any of the following states: Alabama, Arkansas, Delaware, Florida, Georgia,  Kentucky, Louisiana, Maryland, Mississippi, Missouri, North Carolina,  Tennessee, South Carolina, Texas, Virginia |  |
|  |  |  |
|  |  |  |
|  |  |  |
|  |  |  |

Table A2

Descriptive statistics: Cross-sectional data from 2014

| Statistic | N | Mean | St. Dev. | Min | Max |
| --- | --- | --- | --- | --- | --- |
| Life expectancy at birth | 2,830 | 77.7 | 2.4 | 67.6 | 86.8 |
| Probability of death ages 25–45 (%) | 2,830 | 3.1 | 0.9 | 1.2 | 8.3 |
| Probability of death ages 45–65 (%) | 2,830 | 13.3 | 3.1 | 4.8 | 32.6 |
| Incarceration rate per 100,000 population | 2,107 | 313 | 165 | 21.9 | 732 |
| Violent crime rate per 100,000 population | 2,572 | 237 | 194 | 0.0 | 2,569 |
| Median household income ($) | 2,830 | 46,515 | 11,427 | 22,640 | 108,477 |
| Unemployment rate | 2,830 | 6.3 | 2.2 | 1.2 | 24.0 |
| Labor force participation rate | 2,830 | 0.6 | 0.1 | 0.3 | 0.9 |
| Poverty rate | 2,830 | 0.1 | 0.1 | 0.02 | 0.5 |
| Absolute income mobility | 2,673 | 47.5 | 6.1 | 25.1 | 68.3 |
| Income inequality | 2,830 | 0.4 | 0.1 | 0.2 | 0.8 |
| High school graduation rate | 2,830 | 0.9 | 0.1 | 0.5 | 1.0 |
| Fraction African-Americans | 2,830 | 0.1 | 0.1 | 0.002 | 0.9 |
| Fraction Hispanics | 2,830 | 0.1 | 0.1 | 0.004 | 1.0 |
| Fraction other ethnic minority | 2,830 | 0.04 | 0.1 | 0.001 | 0.9 |
| Racial segregation | 2,830 | 0.1 | 0.1 | 0.0 | 0.7 |
| Percentage uninsured | 2,830 | 18.4 | 5.5 | 3.6 | 41.4 |

Notes: All variables, listed in the first column, are measured at the county level in the year 2014. The second column lists the number of observations. The unemployment rate variable is taken from the U.S. Bureau of Labor Statistics. All other variables are from the Opportunity Insights database. Absolute income mobility is measured as the county-level percentage of children who earn more than their parents in 2014. Income inequality is measured as the county-level Gini index within the bottom 99 percent of the income distribution. Racial segregation is measured as residential segregation by race. The final variable measures the percentage of the county population without health insurance. The sources of all remaining variables are listed in A3 below.

Table A3

Descriptive statistics: Panel data from 1983–2014

| Statistic | N | Mean | St. Dev. | Min | Max |
| --- | --- | --- | --- | --- | --- |
| Life expectancy at birth | 59,153 | 76.2 | 2.3 | 66.2 | 86.8 |
| Probability of death ages 25–45 (%) | 59,153 | 3.3 | 0.9 | 1.2 | 10.2 |
| Probability of death ages 45–65 (%) | 59,153 | 14.5 | 3.0 | 4.8 | 32.6 |
| Incarceration rate per 100,000 population | 59,153 | 250.1 | 158.6 | 6.0 | 732.1 |
| Violent crime rate per 100,000 population | 59,153 | 281 | 260 | 0.0 | 3,894 |
| Median household income ($) | 59,153 | 47,533 | 11,853 | 17,583 | 125,705 |
| Fraction African-Americans | 59,153 | 0.1 | 0.1 | 0.0 | 0.9 |

Notes: All variables, listed in the first column, are measured at the county level. The second column lists the number of observed county-years. The three outcome variables—life expectancy at birth and the two measures of premature mortality risk—are taken from the Institute for Health Metrics and Evaluation U.S. Health Map database. The incarceration rate is per 100,000 population aged 16–64 and is constructed by the Vera Institute of Justice. The measure of violent crime is extracted from the Federal Bureau of Investigation’s Uniform Crime Reporting Program. The measure of the county fraction of African-Americans is taken from the U.S. Census Bureau.

Table A4

Mean covariate balance obtained from coarsened exact matching

|  | Treated | Control | Difference | Improvement |
| --- | --- | --- | --- | --- |
| Distance | 0*.*47 | 0*.*46 | 0.01 | 96% |
| Violent crime rate | 223 | 212 | 11 | 93% |
| Median household income ($) | 4*,*4095 | 4*,*4171 | -76 | 99% |
| High school graduation rate | 0*.*73 | 0*.*73 | -0.0006 | 99% |
| Fraction African-Americans | 0*.*08 | 0*.*08 | 0.001 | 99% |
| Fraction Hispanics | 0*.*04 | 0*.*03 | 0.003 | 93% |
| Fraction other ethnicity | 0*.*014 | 0*.*014 | 0.0005 | 40% |

Notes: Results from applying a coarsened exact matching algorithm to time-averaged versions of the variables presented in the Hypotheses, Data, and Methods section. The algorithm produces a matched data set using a dichotomous treatment indicator of above versus below mean exposure to incarceration. The table displays matched covariate levels in the control and treatment groups, as well as the percentage balance improvement as a result of matching.

Table A5

Cross-sectional regression model of life expectancy at birth

| Control variable | Control coefficient | Incarceration coefficient |
| --- | --- | --- |
| Unemployment rate | −1.30%*** | −0.74%*** |
|  | (0.09) | (0.05) |
| Poverty rate | −1.29%*** | −0.74%*** |
|  | (0.08) | (0.05) |
| Absolute income mobility | 0.66%*** | −0.82%*** |
|  | (0.08) | (0.06) |
| Income inequality | 0.09% | −0.89%*** |
|  | (0.06) | (0.06) |
| Racial segregation | −0.02% | −0.88%*** |
|  | (0.06) | (0.06) |
| Percentage uninsured | −0.51%*** | −0.84%*** |
| (0.10) (0.06) | | |

Notes: The log-transformed outcome variable is life expectancy at birth in the year 2014. Each row is a separate regression wherein the association between incarceration and life expectancy is adjusted for the control variable listed in the first column. All models are also adjusted for state-fixed effects. All regressors are standardised by subtracting the mean and dividing by the standard deviation. Parameter estimates are interpreted as the percentage change in the outcome variable associated with a standard deviation increase in each predictor. Robust standard errors are shown in parentheses below each parameter estimate. Statistical significance levels: **p <* 0*.*05; ***p <* 0*.*01; ****p <* 0*.*001.

Table A6

Cross-sectional regression model of premature mortality risk ages 25–45

Control variable Control coefficient Incarceration coefficient

| Unemployment rate | 13.8%*** | 6.6%*** |
| --- | --- | --- |
|  | (0.9) | (0.5) |
| Poverty rate | 13.9%*** | 6.7%*** |
|  | (0.7) | (0.5) |
| Absolute income mobility | −6.9%*** | 7.4%*** |
|  | (0.8) | (0.6) |
| Income inequality | 1.4%* | 7.9%*** |
|  | (0.6) | (0.6) |
| Racial segregation | 1.2%* | 8.0%*** |
|  | (0.6) | (0.6) |
| Percentage uninsured | 10.9%*** | 7.4%*** |
|  | (1.0) | (0.5) |

Notes: The log-transformed outcome variable is the probability of death between the ages of 25 and 45 in the year 2014. Each row is a separate regression wherein the association between incarceration and premature mortality risk is adjusted for the control variable listed in the first column. All models are also adjusted for state-fixed effects. All regressors are standardised by subtracting the mean and dividing by the standard deviation. Parameter estimates are interpreted as the percentage change in the outcome variable associated with a standard deviation increase in each predictor. Robust standard errors are shown in parentheses below each parameter estimate. Statistical significance levels: **p <* 0*.*05; ***p <* 0*.*01; ****p <* 0*.*001.

Table A7

Cross-sectional regression model of premature mortality risk ages 45–65

Control variable Control coefficient Incarceration coefficient

| Unemployment rate | 9.7%*** | 6.3%*** |
| --- | --- | --- |
|  | (0.7) | (0.4) |
| Poverty rate | 9.9%*** | 6.3%*** |
|  | (0.5) | (0.4) |
| Absolute income mobility | −5.7%*** | 6.8%*** |
|  | (0.6) | (0.5) |
| Income inequality | −0.07% | 7.4%*** |
|  | (0.5) | (0.5) |
| Racial segregation | 0.6% | 7.4%*** |
|  | (0.4) | (0.5) |
| Percentage uninsured | 5.5%*** | 6.9%*** |
|  | (0.8) | (0.4) |

Notes: The log-transformed outcome variable is the probability of death between the ages of 45 and 65 in the year 2014. Each row is a separate regression wherein the association between incarceration and premature mortality risk is adjusted for the control variable listed in the first column. All models are also adjusted for state-fixed effects. All regressors are standardised by subtracting the mean and dividing by the standard deviation. Parameter estimates are interpreted as the percentage change in the outcome variable associated with a standard deviation increase in each predictor. Robust standard errors are shown in parentheses below each parameter estimate. Statistical significance levels: **p <* 0*.*05; ***p <* 0*.*01; ****p <* 0*.*001.
